# The Augusta, Georgia Breast Cancer Survivor Study

**Published:** 2018-06-06

**Authors:** Steven S. Coughlin, Valerie Williams, Nicole Moore, Deborah Bowen, Judith Anglin, Nadine Mansur, Gianluca De Leo

**Affiliations:** 1 Department of Clinical and Digital Health Sciences, Augusta University, Augusta, GA; 2 Research Service, Charlie Norwood Veterans Administration Medical Center, Augusta, GA; 3 Physical Therapy Department, Augusta University, Augusta, GA; 4 University of Washington School of Medicine, Seattle, WA

**Keywords:** African Americans, Breast cancer, Diet, Epidemiologic studies, Hispanics, Nutrition, Physical activity, Quality of life

## Abstract

**Introduction:**

Several studies have provided important information about health conditions and other challenges faced by women diagnosed with breast cancer and how they can improve their quality of life and reduce their risk of cancer recurrence. Although African American and Hispanic breast cancer patients have a poorer survival than their white counterparts, few studies have compared the experiences of African American, Hispanic, and non-Hispanic white breast cancer survivors.

**Objectives:**

To facilitate collaborative studies on breast cancer survivorship in a multicultural population, including future intervention research on nutrition, and physical activity, and clinical substudies.

**Methods:**

This cohort study consists of a postal survey of up to 1,000 women with a history of a breast cancer diagnosis who reside in Augusta-Richmond County, Georgia, USA, and a repeat survey in 4 to 5 years to obtain longitudinal data. The follow-up survey in 4 to 5 years will allow for longitudinal changes in health to be assessed.

**Conclusion:**

The survey will provide a comprehensive picture of the health of breast cancer survivors, across the lifespan, in a large Southern city. A broad range of health issues will be addressed including physical activity, diet, nutrition, personal and family history of cancer, quality-of-life, psychosocial concerns, and beliefs about cancer recurrence risk reduction through lifestyle changes. Through its longitudinal design, the study will also provide important information about changes in physical and mental health as breast cancer survivors advance in age.

## INTRODUCTION

There are currently about 2.5 million breast cancer survivors in the U.S. and the number is likely to continue to increase because of the aging of the population^1^. Several studies conducted over the past few decades have provided important information about health conditions and other challenges faced by women diagnosed with breast cancer and how they can improve their quality of life and reduce their risk of cancer recurrence^2^^-^^14^ Women diagnosed with breast cancer and treated with surgery plus adjuvant therapy have a 5-10 year recurrence risk of 5% to 13%^15^^, ^^16^.

Common side effects of breast cancer treatment include lymphedema, loss of strength, difficulty sleeping, and sexual dysfunction^2^^, ^^17^. Longer term symptoms have also been reported including sleep disturbances, depression, fatigue, and pain^18^^,^^19^ Potential late effects of breast cancer treatment include secondary malignancies, cardiovascular disease, obesity, osteoporosis, injuries through falls, bone fractures, declines in physical function, and other conditions that affect physical and emotional wellbeing^20^^-^^22^. Physical inactivity and poor nutritional status including excessive weight gain can occur following breast cancer treatment which increases risk of breast cancer recurrence, other chronic diseases, and all-cause and breast cancer-related mortality^23^. Many breast cancer survivors also face substancial financial burdens and lower access to healthcare.

Exercise can lower circulating levels of estrogen and potentially reduce tumor proliferation. Only about one-third of breast cancer survivors engage in the recommended level of physical activity and less than 18% to 37% consume the recommended amounts of fruits and vegetables^14^^, ^^24^. Eating a healthy diet that includes adequate fruits and vegetables, whole grains, and little or no red meat protects against several chronic diseases. Consuming an unhealthy diet and physical inactivity increase risk of obesity and non-breast cancer mortality^17^. Poor diet also increases risk of fatigue^25^.

Relatively few studies have compared the experiences of African American, Hispanic, and non-Hispanic white breast cancer survivors^5^^, ^^8^^, ^^26^^-^^29^. Several studies have focused specifically on Hispanic American women^30^^-^^47^. Other studies have focused specifically on African American breast cancer survivors because of their higher risk of more deadly, “triple negative” tumors, poorer survival, and higher rates of obesity^12^^, ^^14^^, ^^24^^, ^^48^^-^^52^. African American breast cancer survivors have been found to be more likely to be obese, less likely to engage in physical activity, and more likely to have decreased physical functioning than other groups of breast cancer survivors^17^. Racial and ethnic minorities often face increased financial barriers to followup care^53^. Low socioeconomic status has been found to be inversely related to health related quality of life among breast cancer survivors^6^.

The objectives of the Augusta, Georgia Breast Cancer Survivor Study (AGBSS) are: 1) to collect baseline information via a postal questionnaire from a multiethnic cohort of breast cancer survivors who reside in Augusta, GA about physical activity, nutrition, medical and psychiatric history, personal and family history of cancer, upper extremity limitation, receipt of physical therapy or occupational therapy, psychosocial concerns, and beliefs about cancer recurrence risk reduction through lifestyle changes; 2) to compare groups of survivors defined by race, Hispanic ethnicity, age categories, stage-at-diagnosis, and years since breast cancer diagnosis; 3) to follow the women longitudinally and invite them to participate in a follow-up survey in 4-5 years; and 4) to facilitate IRB-approved clinical research studies that include subsets of women surveyed as part of the AGBSS.

## MATERIALS AND METHODS

This cohort study consists of a postal survey and a repeat survey in 4 to 5 years to obtain longitudinal data. The follow-up survey in 4 to 5 years will allow for longitudinal changes in health to be assessed.

### Study Population

The study population will consist of up to 1,000 women ages ≥ 18 years with a self-reported diagnosis of breast cancer (i.e., prevalent cases) who currently reside in Augusta, Georgia. We anticipate that about 30% to 40% of the women will be African American and that the majority will be > 60 years of age. In order to ensure adequate numbers for comparisons across groups, women with a Hispanic surname will be over-sampled. Non-institutionalized women (e.g., those who are not in a nursing home) will be eligible to take part in this study if they reside in Georgia and are able to provide informed consent to a postal survey in English. Women’s names and mailing addresses were obtained from a commercial firm that provides health information to members of insurance plans. The women indicated that they were interested in receiving information about breast cancer.

### Data Collection

Data will be collected using postal survey questionnaires. All subjects will have an assigned participant ID number. A sequential mailing protocol will be followed using a modified Dillman method^54^. An advance letter will be mailed to the women by the study principal investigator. The letter will provide information about the study (purpose, potential benefits, and risks) and let them know how they can opt out and not receive further mailings about the study. Three weeks later, a survey consent letter will be mailed to women who have not opted out along with a copy of the survey questionnaire and a pre-addressed, stamped return envelope. The baseline and follow-up survey questionnaires will take about 30 to 45 minutes to complete.

Women who have not opted out or returned a completed questionnaire will be sent a reminder postcard four weeks later, followed four weeks later by a duplicate copy of the survey consent letter, survey questionnaire, and a preaddressed, stamped return envelope. Women who have not opted out or returned a completed questionnaire will be sent a second reminder postcard four weeks later. In about 4 to 5 years, the panel of women who participate in the baseline survey will be re-contacted for a follow-up postal survey or telephone interview. Survey responses will be checked for completeness and then coded and entered into an electronic database. All survey data collected as part of the study will be carefully monitored for completeness. If a respondent returns two copies of the questionnaire, the most complete questionnaire will be selected for inclusion. The quality of the data will be maximized through pre-coded responses and computerized internal consistency checks and range checks of specified values.

Only breast cancer survivors who reside in Augusta-Richmond County, Georgia are included in the computer file. In the first stage of the postal survey, the mailings will be sent to 1,000 potential research participants. Because the response rate to the postal survey is likely to be in the range of 40-60%, it will be necessary to obtain an additional random sample of women included in the computer file of names and mailing addresses in the second phase of the postal survey so that the final study population is about 1,000 participants.

### Measures

Questions about demographic factors and breast cancer diagnosis were obtained from a previous study of breast cancer survivors^8^ A nutrition screening questionnaire was adapted from the Rapid Eating and Activity Assessment for Participants short version^55^ and LEAN eating questionnaire^56^. Questions about physical activity were obtained from a previous study of breast cancer survivors^8^ and the self-administered instrument designed for the Women’s Health Imitative (57). The SF-12 will be used to measure physical activity limitations^58^. Questions about upper extremity function and disability were obtained from the Disabilities of the Arm, Shoulder, and Hand Questionnaire^59^. Questions about breast cancer recurrence risk reduction beliefs and behaviors were obtained from a previous study of breast cancer survivors^9^. The Cancer Problems in Living Scale^60^, as adapted for a previous study of breast cancer survivors^12^, will be used to assess problems that women may encounter as a breast cancer survivor. The survey questionnaire developed for this study is available from the authors by request.

### Data Analyses

The approach that will be taken for statistical analysis of the data is as follows. Initially, crosstabulations of the data will be performed using SAS. Both chi-square and Fisher’s exact tests will be used to examine the statistical significance of observed associations. After crosstabulations and exploratory analyses of the survey data are completed, logistic regression methods will be used to compare groups of breast cancer survivors defined by race, Hispanic ethnicity, age categories, stage-at-diagnosis, years since breast cancer diagnosis, and whether breast cancer reoccurred. Potential confounding factors will be controlled for in these analyses (e.g., age, education). Potential effect modifiers will initially be examined in exploratory crosstabulations of the data and then by including interaction terms in logistic models and performing Log-likelihood ratio tests. Ninety-five percent confidence intervals will be obtained for adjusted odds ratios. Levels of statistical significance will be determined using Wald chi-square tests and Log-likelihood ratio tests. The goodness-of-fit of each model will be examined using the Log-likelihood ratio tests. Potential confounding will be dealt with through stratification and by controlling for study cohort in logistic regression models.

To analyze data on the frequency of medical conditions diagnosed by healthcare providers, crosstabulations of the data will be performed. Chi-square and Fisher’s exact tests will be used to examine the statistical significance of observed associations. Prevalence odds ratios will be obtained with 95% confidence intervals. Additional analyses will be stratified on age categories, stage-at-diagnosis, and years since breast cancer diagnosis. Prevalence odds ratios associated with chronic symptoms and medical conditions will be obtained using logistic regression. These outcomes will be assessed in relation to the study variables of interest using stratified analyses and included in multivariable models as appropriate. Demographic variables (age category, race, Hispanic ethnicity, and education) will be included in the models.

Health changes over time will be examined using baseline and follow-up data. These analyses will be limited to women who participated in both the baseline and followup surveys. Crosstabulations of the data will initially be performed. Chi-square and Fisher’s exact tests will be used to examine the statistical significance of observed temporal associations. Prevalence odds ratios associated with chronic symptoms and chronic medical conditions will be obtained using logistic regression. Demographic variables (age at baseline, race, Hispanic ethnicity, education) will be included in the models as appropriate. Additional comparisons will be made with data from the National Health and Nutrition Examination Survey.

### Sample Size Calculations

Sample size calculations were carried out based upon an array of assumptions, taking into account likely attrition. Based upon a literature review, the percentage of women who report a history of outcomes of interest will likely vary widely between 5-50%. Sample size calculations were carried out for a two-tailed test on proportions (P_1_, P_2_). For a two-tailed test on proportions, with a type I error rate of 5% and power of .80, and where P_1_ = .15 and P_2_=.30, 134 women per group (or 268 women total) would be needed to detect this difference. For ordinal variables, the statistical power to detect meaningful differences across groups will be greater.

### Limitations

Although validation studies have shown that people are able to accurately report information about medical conditions and health care utilization, some self-reported information may be affected by misclassification bias. Because one of the goals of the Augusta Georgia Breast Cancer Survivor Study is to establish a cohort of women who can be invited to participate in health intervention and clinical research studies, it may be possible to validate some medical conditions and exposures at the time of these studies. The use of a sample of women interested in obtaining further information about breast cancer is a potential source of bias. Including long-term survivors will allow study of long term health effects, but information on early effects may be biased because of recall bias, and losses due to mortality. However, recently diagnosed survivors will also be included in the initial survey. In the follow-up study, some bias may occur due to losses to follow-up or because some women die in the time interval. In the follow-up study, the response rate may be less than 80%. Although the generalizability of the results to other populations may be uncertain, the study design was selected in order to pave the way for clinical sub-studies involving breast cancer survivors who live in Augusta-Richmond County, in proximity to the Augusta University Health Sciences Campus.

### Human Subjects

The study protocol has been submitted for review and approval by the Augusta University IRB. There are no known risks to participants in this survey other than potential, minor psychological distress. The potential benefits of the study are societal in nature and include obtaining new information about the health and health care utilization of breast cancer survivors. There is no direct benefit to participants in this study. Personal identifiers will be removed from study-related health information and kept in a locking cabinet in the principal investigator’s office at Augusta University. Paper-based records will be kept under lock and key and will only be accessible to personnel involved with this study. Data files will be kept on password protected office computers at Augusta University. Only members of the study team will have access to the data.

## CONCLUSIONS

The Augusta, Georgia Breast Cancer Survivor Study will address the need for information about the comprehensive health of multicultural women diagnosed with breast cancer who live in a large city in the Southern U.S. The study is likely to contribute importantly to our understanding of racial and ethnic differences in key breast cancer survivorship concerns across the lifespan from young adulthood to older age. A broad range of women’s health issues will be addressed including physical activity, nutrition, personal and family history of cancer, quality-of-life, psychosocial concerns, and beliefs about cancer recurrence risk reduction through lifestyle changes. Through its longitudinal design, the study will provide important information about changes in physical and mental health as breast cancer survivors age.

A particular goal of the study is to establish a cohort of breast cancer survivors who can be invited to participate in IRB-approved health intervention and clinical research studies (e.g., clinical substudies on diet and nutrition, and on upper extremity limitations and lymphedema) that include subsets of women surveyed as part of the Augusta Georgia Breast Cancer Survivor Study. Potential topics for these sub-studies include chronic pain, lymphedema, physical limitations; and chronic conditions that are prevalent in both cancer survivors and the general population as people reach middle age or older age (for example, diabetes, cardiovascular disease).
